# Supplementary figures and images for: Axon tension regulates fasciculation/defasciculation through the control of axon shaft zippering
Source: eLife. 2017 Apr 19;6:e19907. doi: 10.7554/eLife.19907 (PMC5478281; doi:10.7554/eLife.19907)

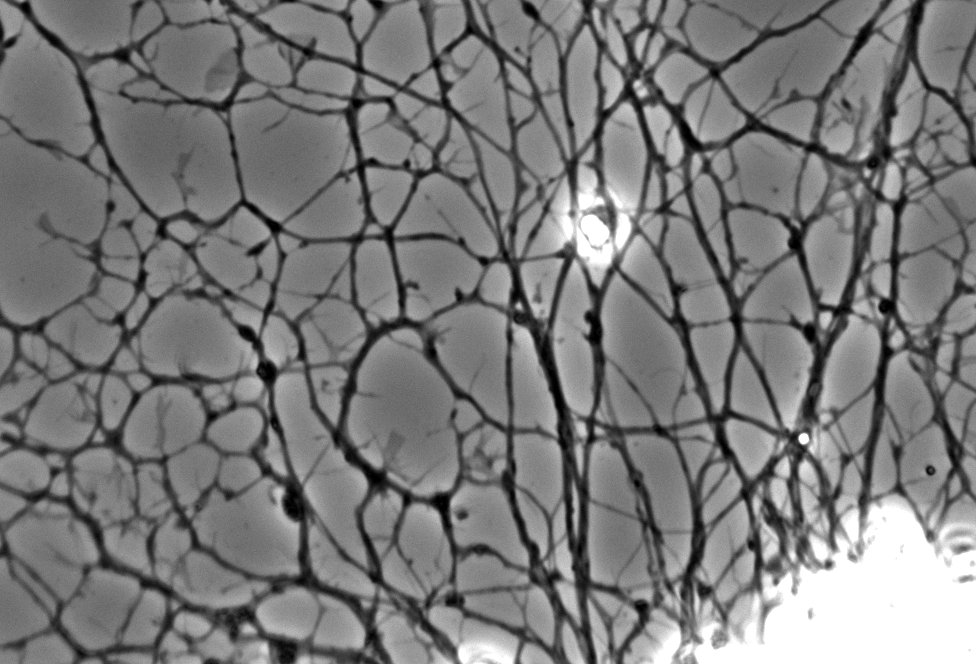

Supplement: Figure 6—source data 8. — The archive contains analyzed frames (TIFF format) and corresponding segmentation selection data (ImageJ-generated ZIP archives). Data can be displayed using ImageJ, please refer to Materials and methods. DOI: http://dx.doi.org/10.7554/eLife.19907.027 [file elife-19907-fig6-data8.zip › Figure_6_source_data_8/video13-1.tif]

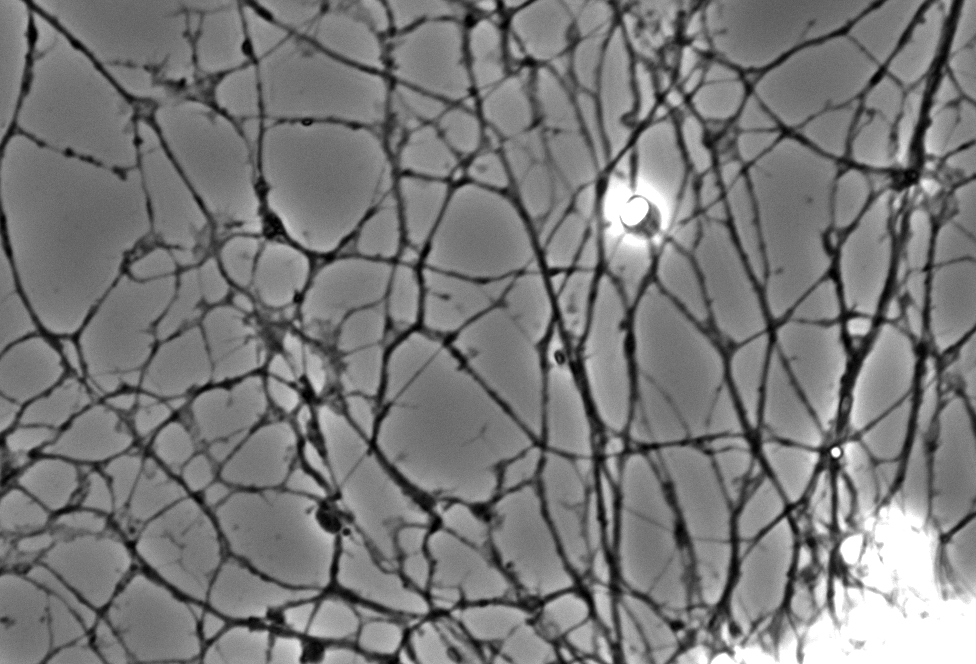

Supplement: Figure 6—source data 8. — The archive contains analyzed frames (TIFF format) and corresponding segmentation selection data (ImageJ-generated ZIP archives). Data can be displayed using ImageJ, please refer to Materials and methods. DOI: http://dx.doi.org/10.7554/eLife.19907.027 [file elife-19907-fig6-data8.zip › Figure_6_source_data_8/video13-100.tif]

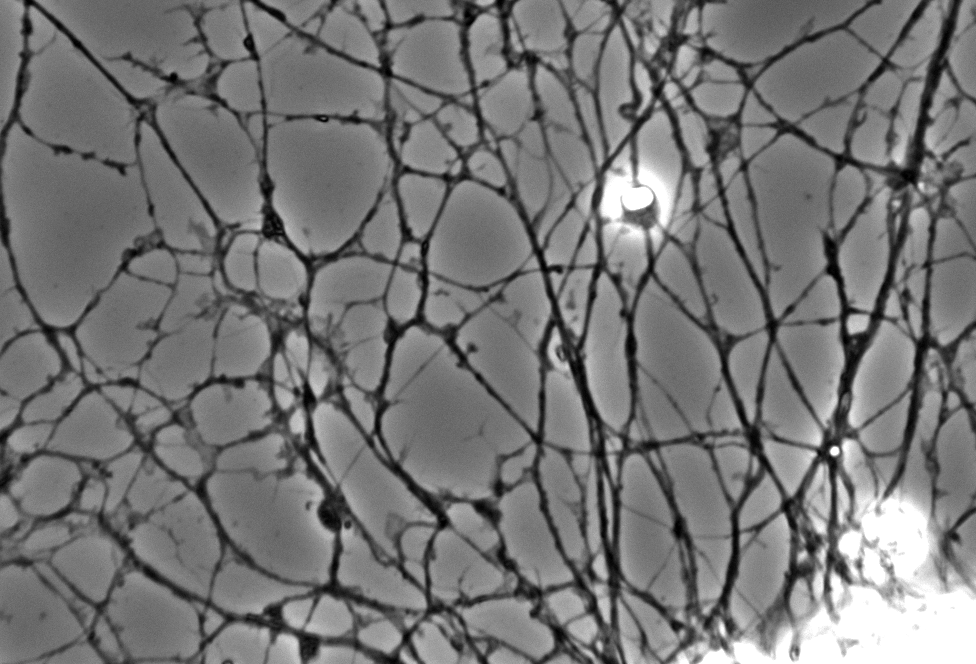

Supplement: Figure 6—source data 8. — The archive contains analyzed frames (TIFF format) and corresponding segmentation selection data (ImageJ-generated ZIP archives). Data can be displayed using ImageJ, please refer to Materials and methods. DOI: http://dx.doi.org/10.7554/eLife.19907.027 [file elife-19907-fig6-data8.zip › Figure_6_source_data_8/video13-120.tif]

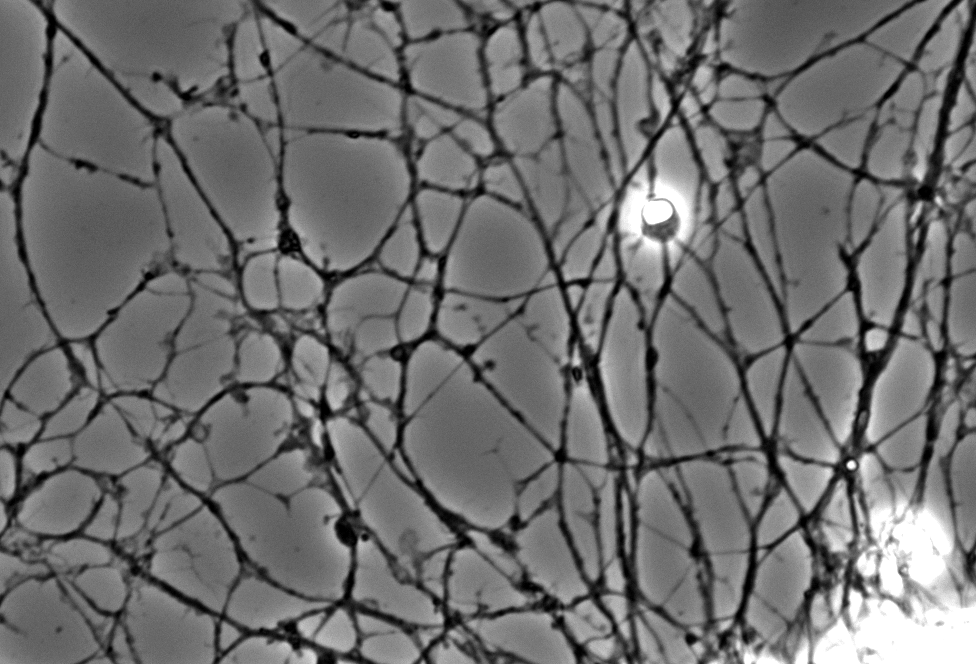

Supplement: Figure 6—source data 8. — The archive contains analyzed frames (TIFF format) and corresponding segmentation selection data (ImageJ-generated ZIP archives). Data can be displayed using ImageJ, please refer to Materials and methods. DOI: http://dx.doi.org/10.7554/eLife.19907.027 [file elife-19907-fig6-data8.zip › Figure_6_source_data_8/video13-140.tif]

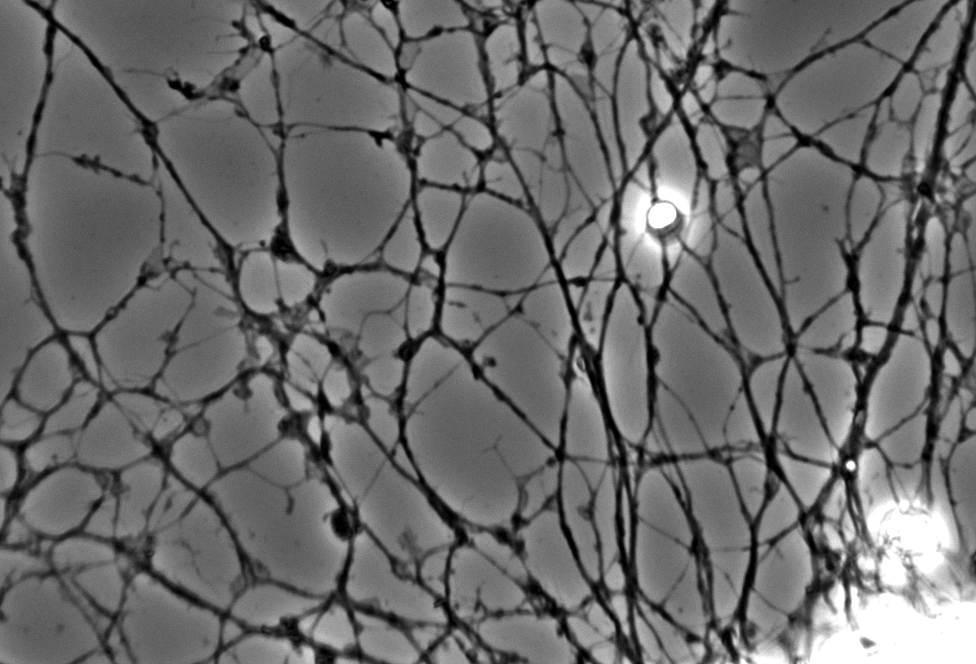

Supplement: Figure 6—source data 8. — The archive contains analyzed frames (TIFF format) and corresponding segmentation selection data (ImageJ-generated ZIP archives). Data can be displayed using ImageJ, please refer to Materials and methods. DOI: http://dx.doi.org/10.7554/eLife.19907.027 [file elife-19907-fig6-data8.zip › Figure_6_source_data_8/video13-160.tif]

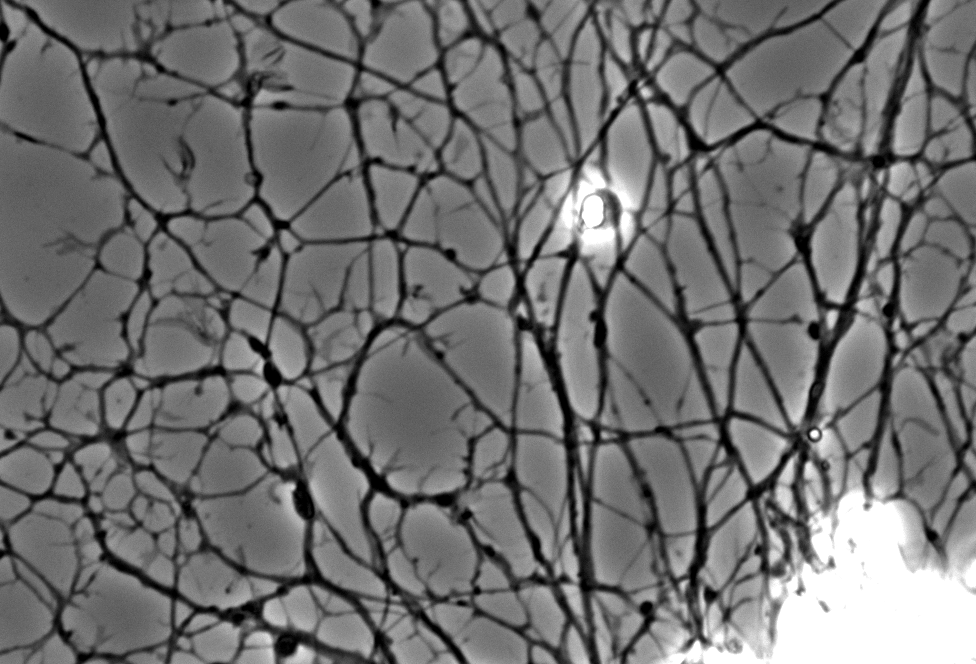

Supplement: Figure 6—source data 8. — The archive contains analyzed frames (TIFF format) and corresponding segmentation selection data (ImageJ-generated ZIP archives). Data can be displayed using ImageJ, please refer to Materials and methods. DOI: http://dx.doi.org/10.7554/eLife.19907.027 [file elife-19907-fig6-data8.zip › Figure_6_source_data_8/video13-20.tif]

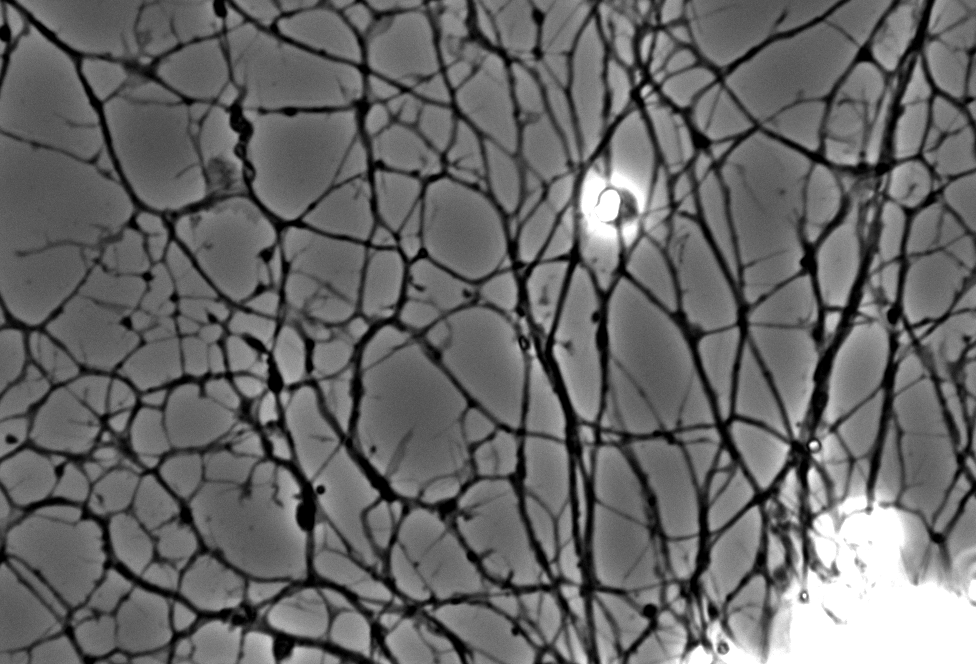

Supplement: Figure 6—source data 8. — The archive contains analyzed frames (TIFF format) and corresponding segmentation selection data (ImageJ-generated ZIP archives). Data can be displayed using ImageJ, please refer to Materials and methods. DOI: http://dx.doi.org/10.7554/eLife.19907.027 [file elife-19907-fig6-data8.zip › Figure_6_source_data_8/video13-40.tif]

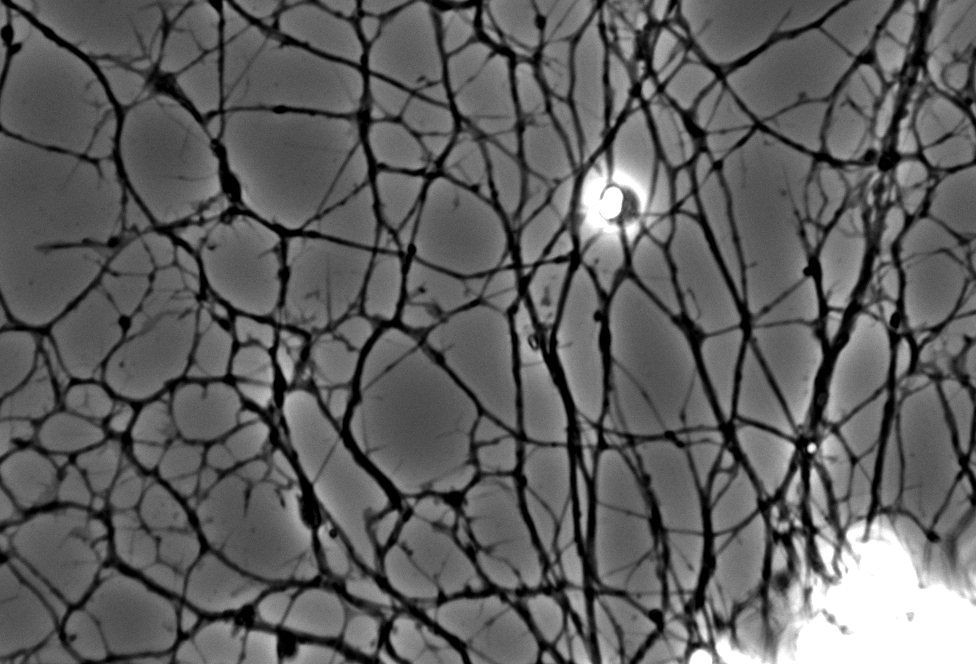

Supplement: Figure 6—source data 8. — The archive contains analyzed frames (TIFF format) and corresponding segmentation selection data (ImageJ-generated ZIP archives). Data can be displayed using ImageJ, please refer to Materials and methods. DOI: http://dx.doi.org/10.7554/eLife.19907.027 [file elife-19907-fig6-data8.zip › Figure_6_source_data_8/video13-60.tif]

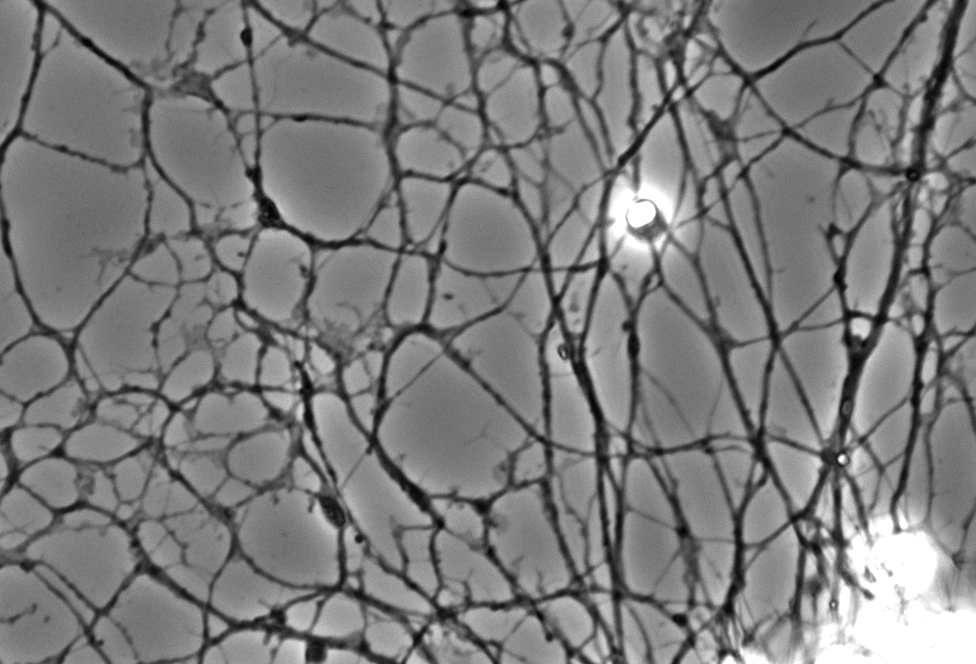

Supplement: Figure 6—source data 8. — The archive contains analyzed frames (TIFF format) and corresponding segmentation selection data (ImageJ-generated ZIP archives). Data can be displayed using ImageJ, please refer to Materials and methods. DOI: http://dx.doi.org/10.7554/eLife.19907.027 [file elife-19907-fig6-data8.zip › Figure_6_source_data_8/video13-70.tif]

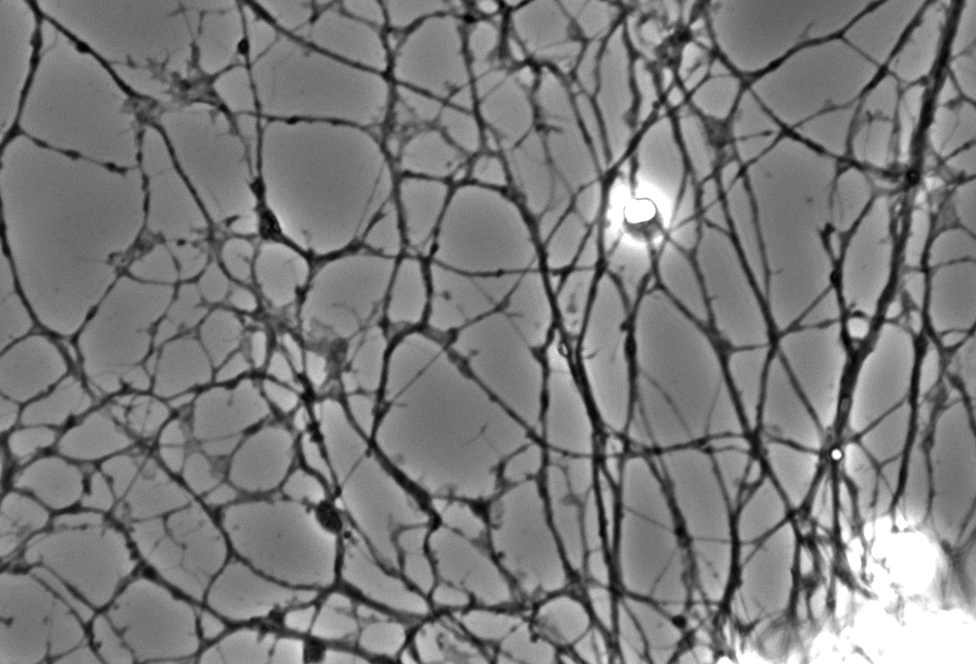

Supplement: Figure 6—source data 8. — The archive contains analyzed frames (TIFF format) and corresponding segmentation selection data (ImageJ-generated ZIP archives). Data can be displayed using ImageJ, please refer to Materials and methods. DOI: http://dx.doi.org/10.7554/eLife.19907.027 [file elife-19907-fig6-data8.zip › Figure_6_source_data_8/video13-80.tif]

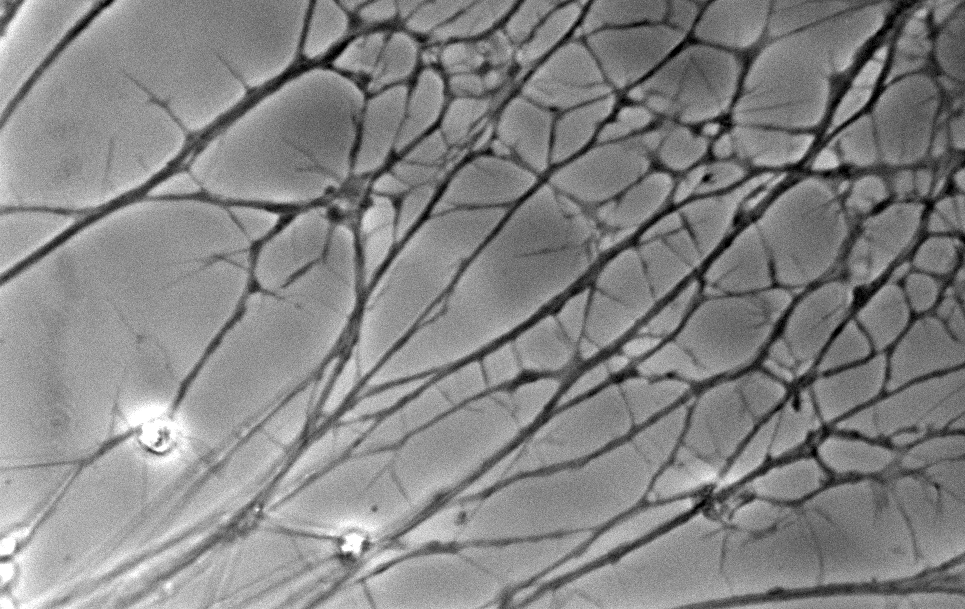

Supplement: Figure 6—source data 8. — The archive contains analyzed frames (TIFF format) and corresponding segmentation selection data (ImageJ-generated ZIP archives). Data can be displayed using ImageJ, please refer to Materials and methods. DOI: http://dx.doi.org/10.7554/eLife.19907.027 [file elife-19907-fig6-data8.zip › Figure_6_source_data_8/video14-1.tif]

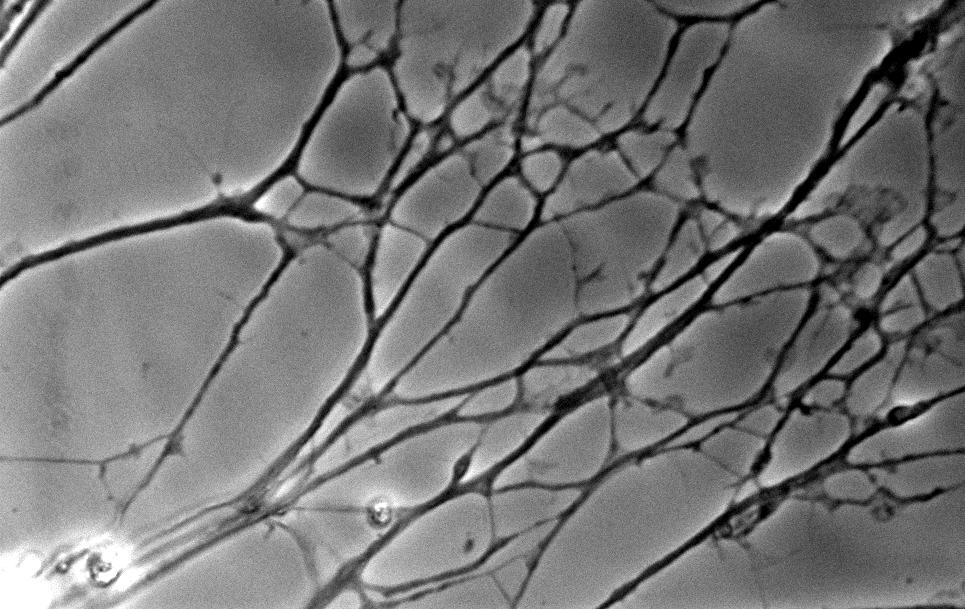

Supplement: Figure 6—source data 8. — The archive contains analyzed frames (TIFF format) and corresponding segmentation selection data (ImageJ-generated ZIP archives). Data can be displayed using ImageJ, please refer to Materials and methods. DOI: http://dx.doi.org/10.7554/eLife.19907.027 [file elife-19907-fig6-data8.zip › Figure_6_source_data_8/video14-100.tif]

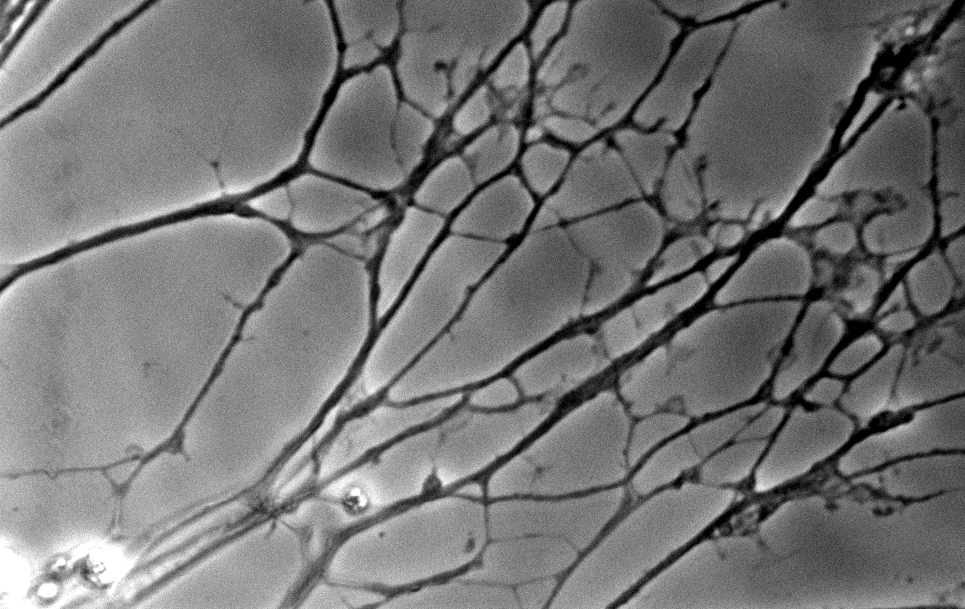

Supplement: Figure 6—source data 8. — The archive contains analyzed frames (TIFF format) and corresponding segmentation selection data (ImageJ-generated ZIP archives). Data can be displayed using ImageJ, please refer to Materials and methods. DOI: http://dx.doi.org/10.7554/eLife.19907.027 [file elife-19907-fig6-data8.zip › Figure_6_source_data_8/video14-120.tif]

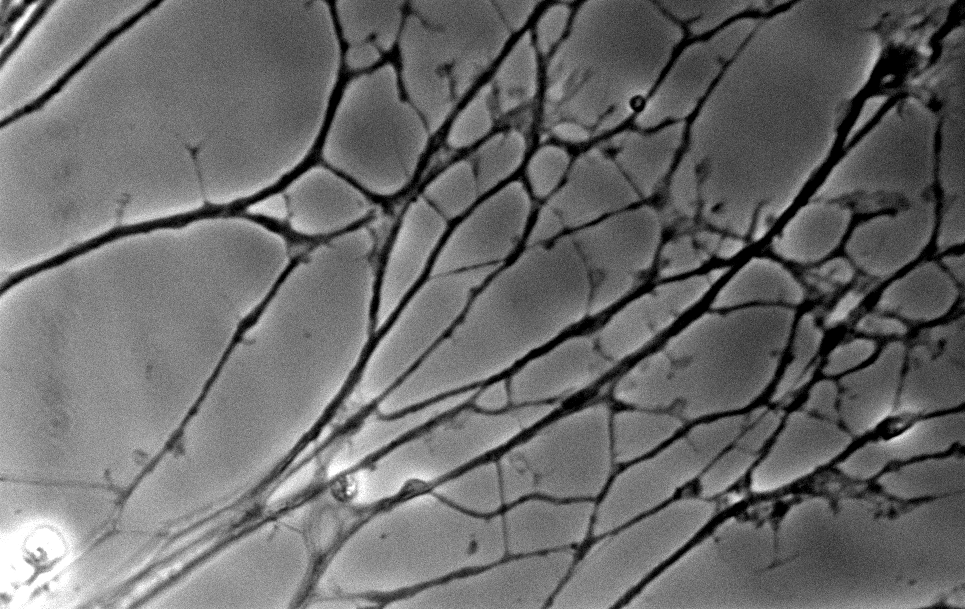

Supplement: Figure 6—source data 8. — The archive contains analyzed frames (TIFF format) and corresponding segmentation selection data (ImageJ-generated ZIP archives). Data can be displayed using ImageJ, please refer to Materials and methods. DOI: http://dx.doi.org/10.7554/eLife.19907.027 [file elife-19907-fig6-data8.zip › Figure_6_source_data_8/video14-150.tif]

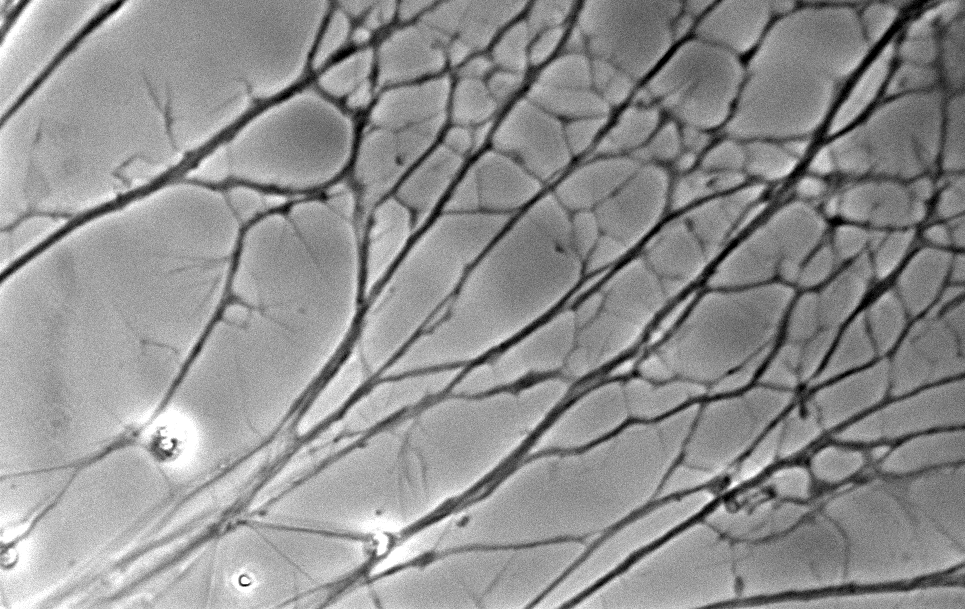

Supplement: Figure 6—source data 8. — The archive contains analyzed frames (TIFF format) and corresponding segmentation selection data (ImageJ-generated ZIP archives). Data can be displayed using ImageJ, please refer to Materials and methods. DOI: http://dx.doi.org/10.7554/eLife.19907.027 [file elife-19907-fig6-data8.zip › Figure_6_source_data_8/video14-20.tif]

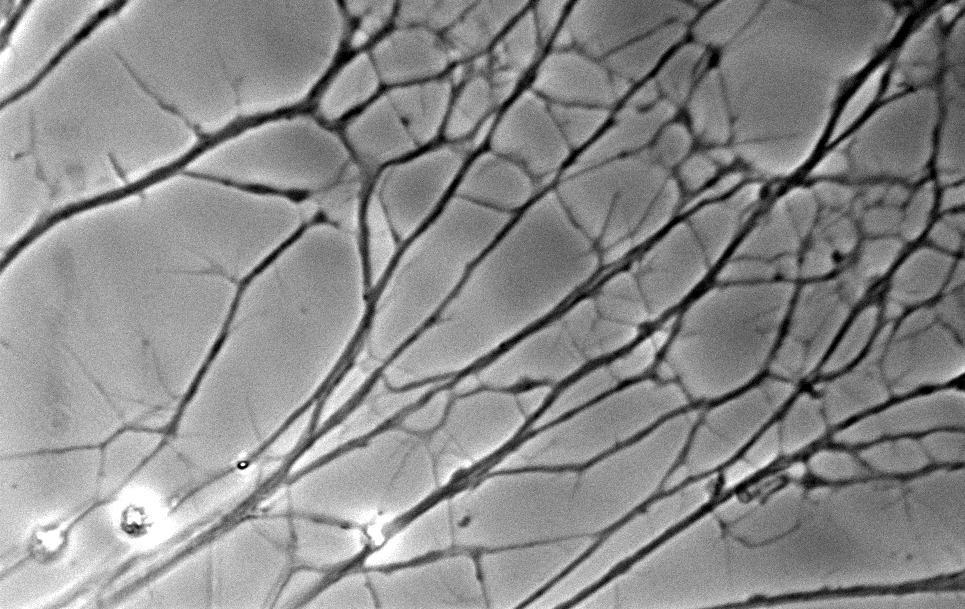

Supplement: Figure 6—source data 8. — The archive contains analyzed frames (TIFF format) and corresponding segmentation selection data (ImageJ-generated ZIP archives). Data can be displayed using ImageJ, please refer to Materials and methods. DOI: http://dx.doi.org/10.7554/eLife.19907.027 [file elife-19907-fig6-data8.zip › Figure_6_source_data_8/video14-40.tif]

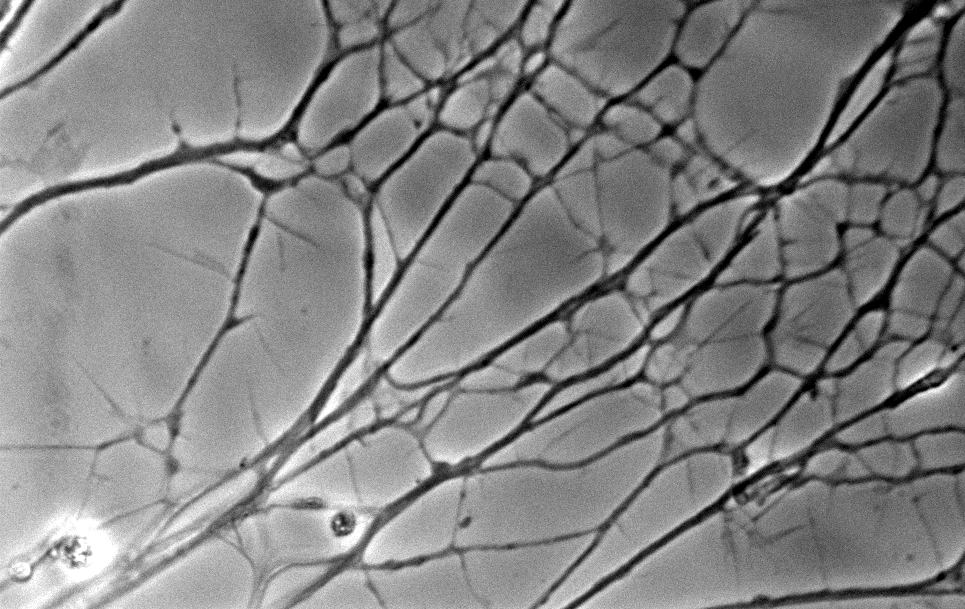

Supplement: Figure 6—source data 8. — The archive contains analyzed frames (TIFF format) and corresponding segmentation selection data (ImageJ-generated ZIP archives). Data can be displayed using ImageJ, please refer to Materials and methods. DOI: http://dx.doi.org/10.7554/eLife.19907.027 [file elife-19907-fig6-data8.zip › Figure_6_source_data_8/video14-60.tif]

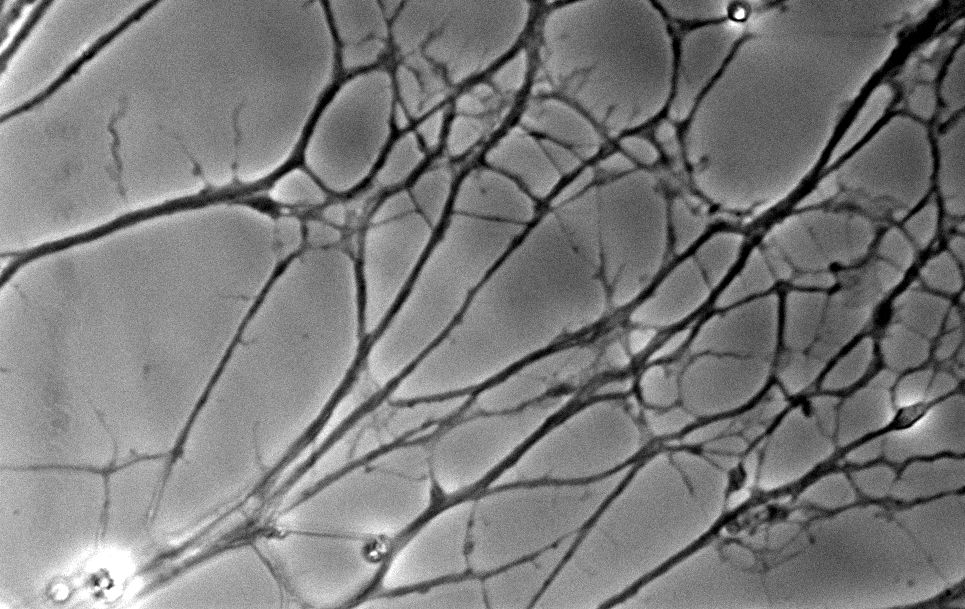

Supplement: Figure 6—source data 8. — The archive contains analyzed frames (TIFF format) and corresponding segmentation selection data (ImageJ-generated ZIP archives). Data can be displayed using ImageJ, please refer to Materials and methods. DOI: http://dx.doi.org/10.7554/eLife.19907.027 [file elife-19907-fig6-data8.zip › Figure_6_source_data_8/video14-70.tif]

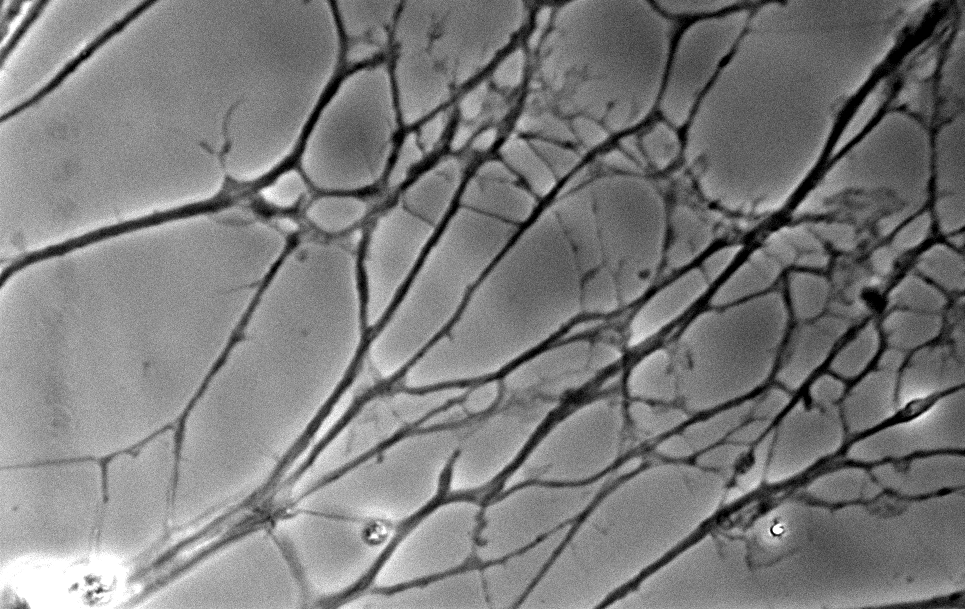

Supplement: Figure 6—source data 8. — The archive contains analyzed frames (TIFF format) and corresponding segmentation selection data (ImageJ-generated ZIP archives). Data can be displayed using ImageJ, please refer to Materials and methods. DOI: http://dx.doi.org/10.7554/eLife.19907.027 [file elife-19907-fig6-data8.zip › Figure_6_source_data_8/video14-80.tif]

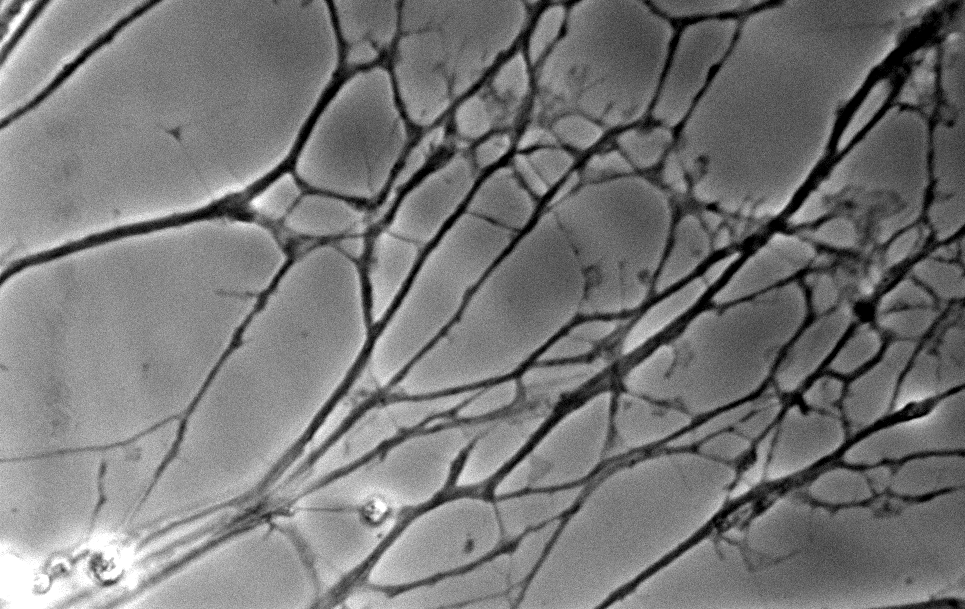

Supplement: Figure 6—source data 8. — The archive contains analyzed frames (TIFF format) and corresponding segmentation selection data (ImageJ-generated ZIP archives). Data can be displayed using ImageJ, please refer to Materials and methods. DOI: http://dx.doi.org/10.7554/eLife.19907.027 [file elife-19907-fig6-data8.zip › Figure_6_source_data_8/video14-90.tif]

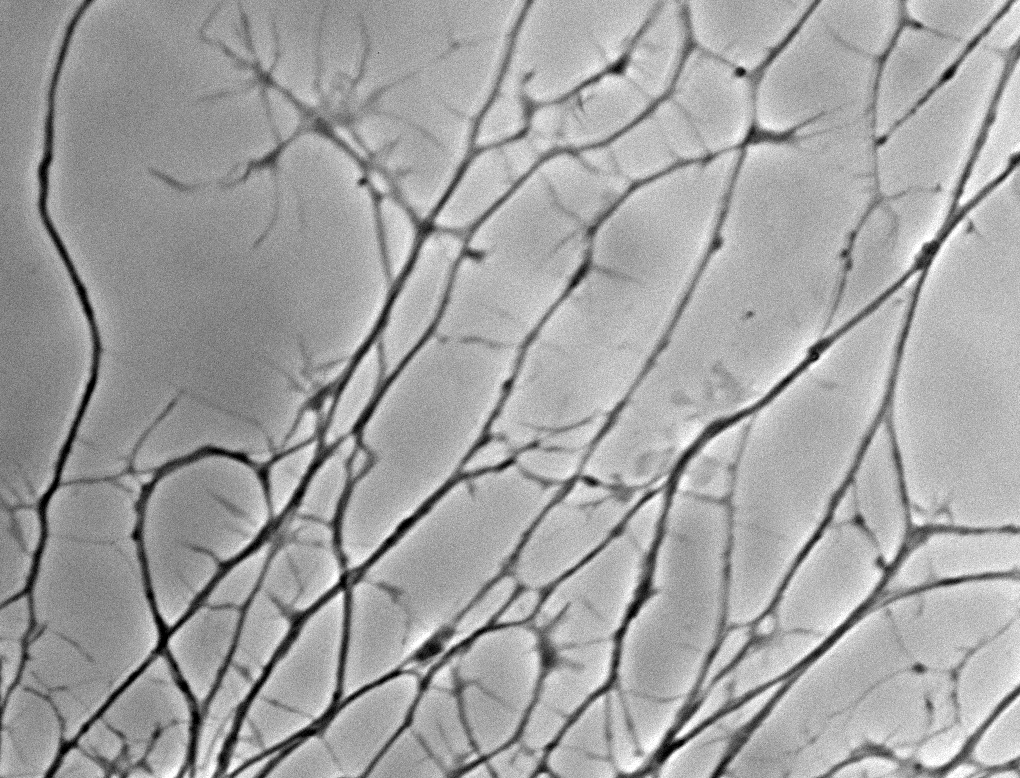

Supplement: Figure 6—source data 8. — The archive contains analyzed frames (TIFF format) and corresponding segmentation selection data (ImageJ-generated ZIP archives). Data can be displayed using ImageJ, please refer to Materials and methods. DOI: http://dx.doi.org/10.7554/eLife.19907.027 [file elife-19907-fig6-data8.zip › Figure_6_source_data_8/video15-1.tif]

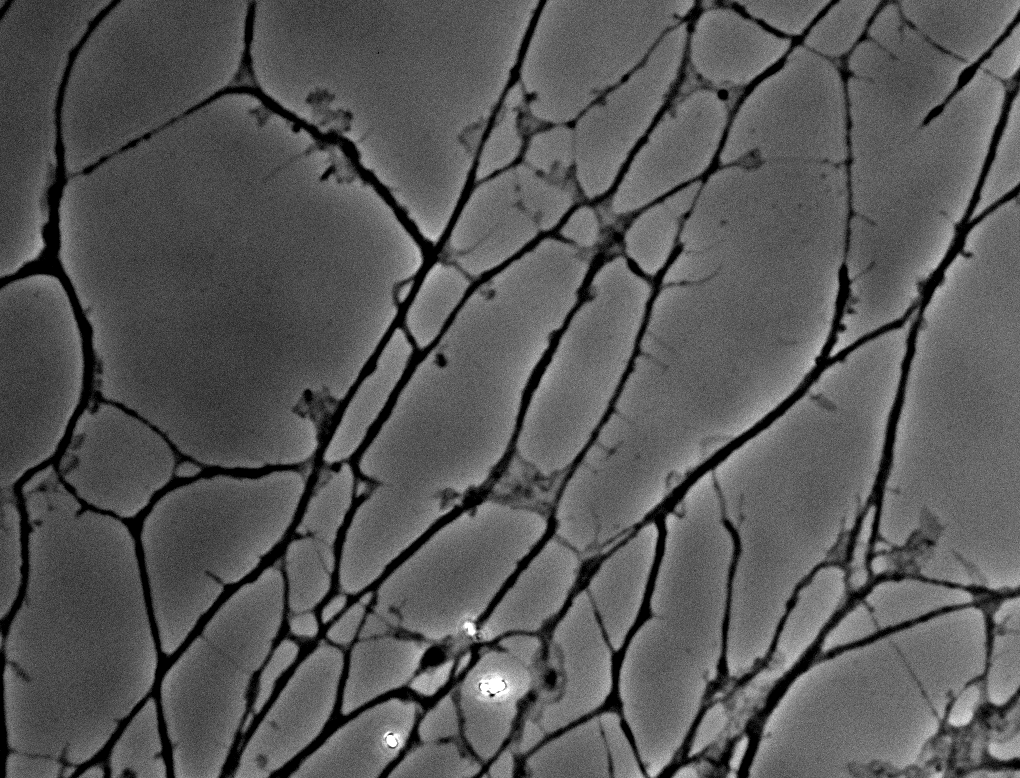

Supplement: Figure 6—source data 8. — The archive contains analyzed frames (TIFF format) and corresponding segmentation selection data (ImageJ-generated ZIP archives). Data can be displayed using ImageJ, please refer to Materials and methods. DOI: http://dx.doi.org/10.7554/eLife.19907.027 [file elife-19907-fig6-data8.zip › Figure_6_source_data_8/video15-100.tif]

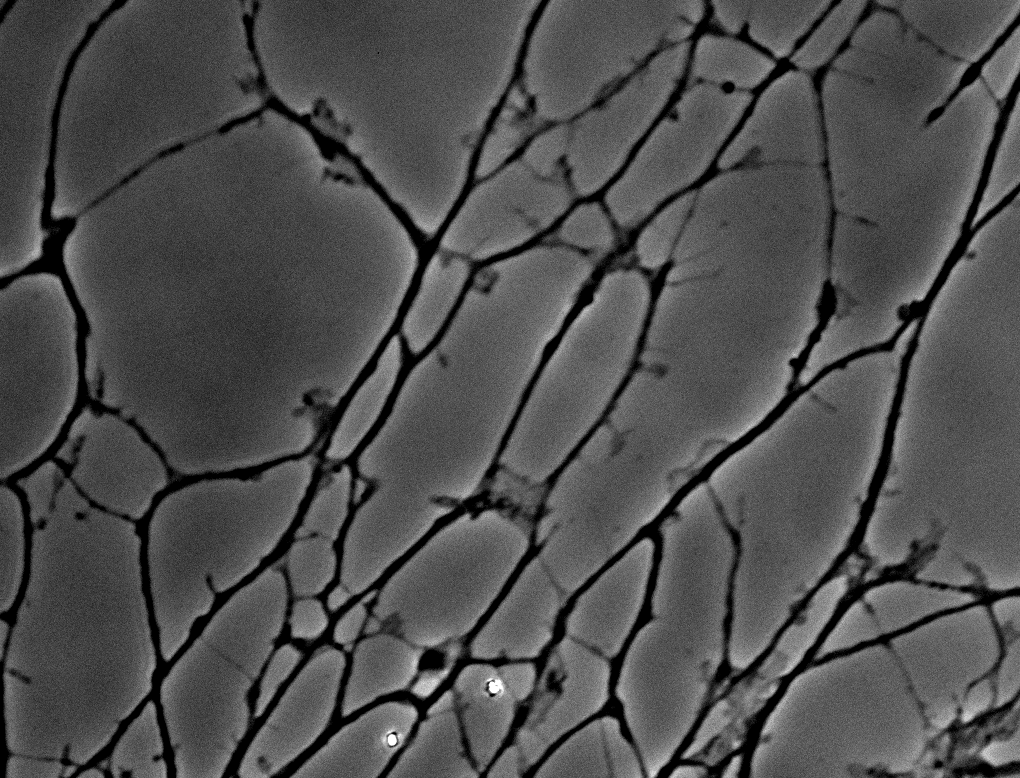

Supplement: Figure 6—source data 8. — The archive contains analyzed frames (TIFF format) and corresponding segmentation selection data (ImageJ-generated ZIP archives). Data can be displayed using ImageJ, please refer to Materials and methods. DOI: http://dx.doi.org/10.7554/eLife.19907.027 [file elife-19907-fig6-data8.zip › Figure_6_source_data_8/video15-120.tif]

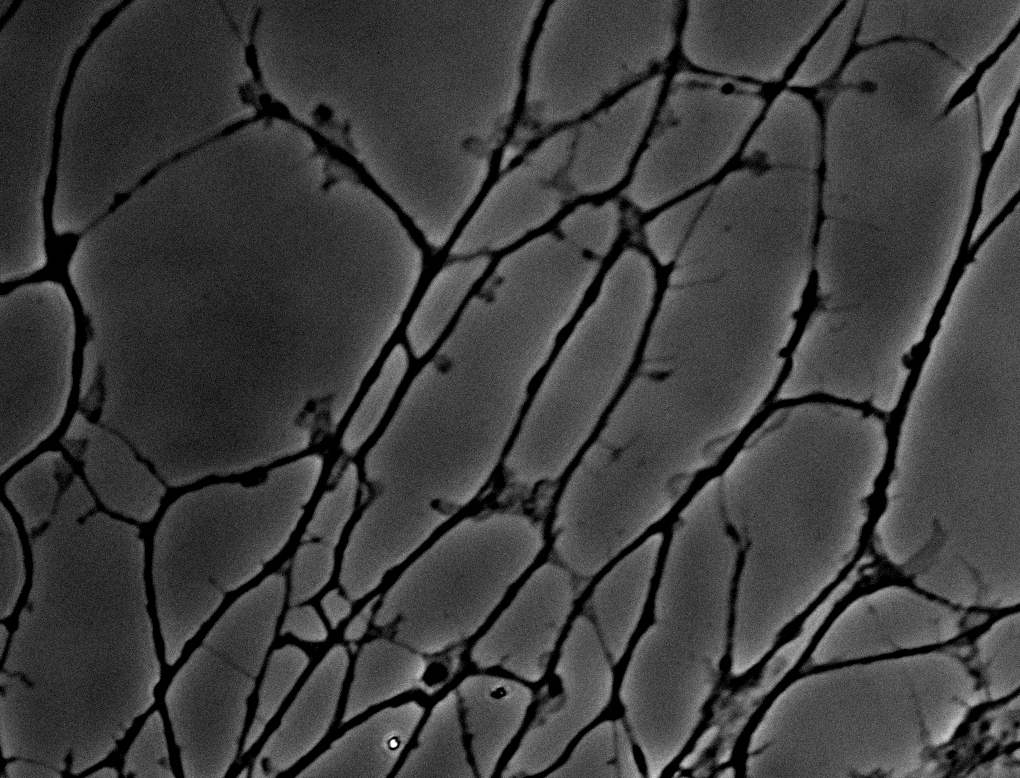

Supplement: Figure 6—source data 8. — The archive contains analyzed frames (TIFF format) and corresponding segmentation selection data (ImageJ-generated ZIP archives). Data can be displayed using ImageJ, please refer to Materials and methods. DOI: http://dx.doi.org/10.7554/eLife.19907.027 [file elife-19907-fig6-data8.zip › Figure_6_source_data_8/video15-150.tif]

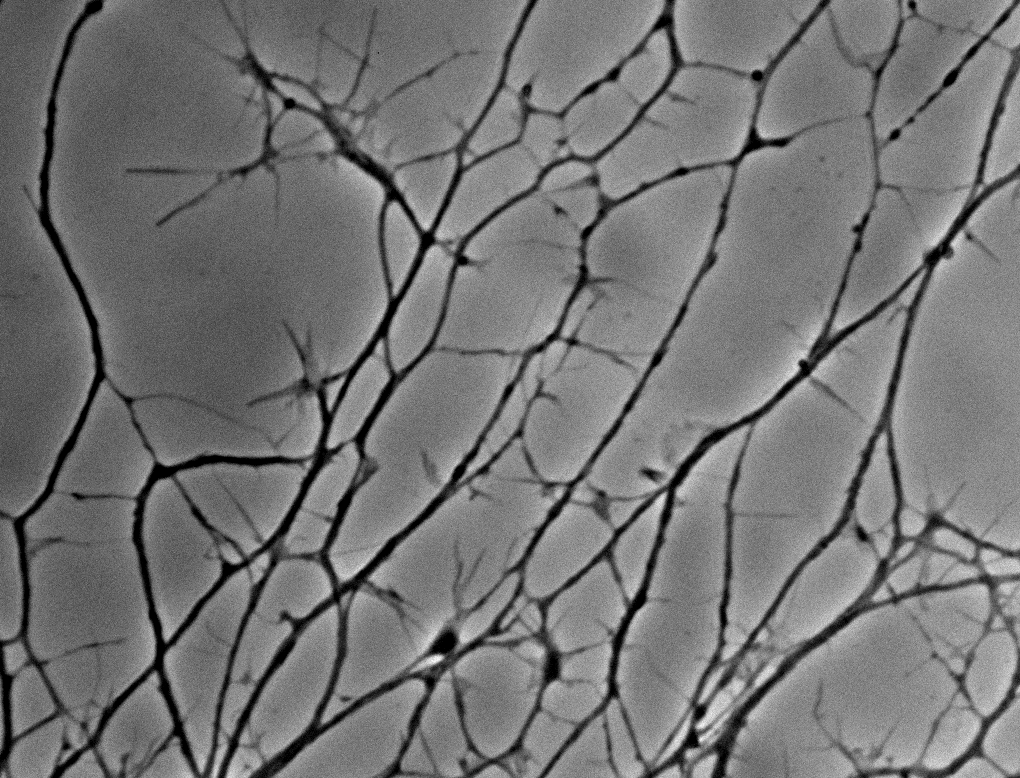

Supplement: Figure 6—source data 8. — The archive contains analyzed frames (TIFF format) and corresponding segmentation selection data (ImageJ-generated ZIP archives). Data can be displayed using ImageJ, please refer to Materials and methods. DOI: http://dx.doi.org/10.7554/eLife.19907.027 [file elife-19907-fig6-data8.zip › Figure_6_source_data_8/video15-30.tif]

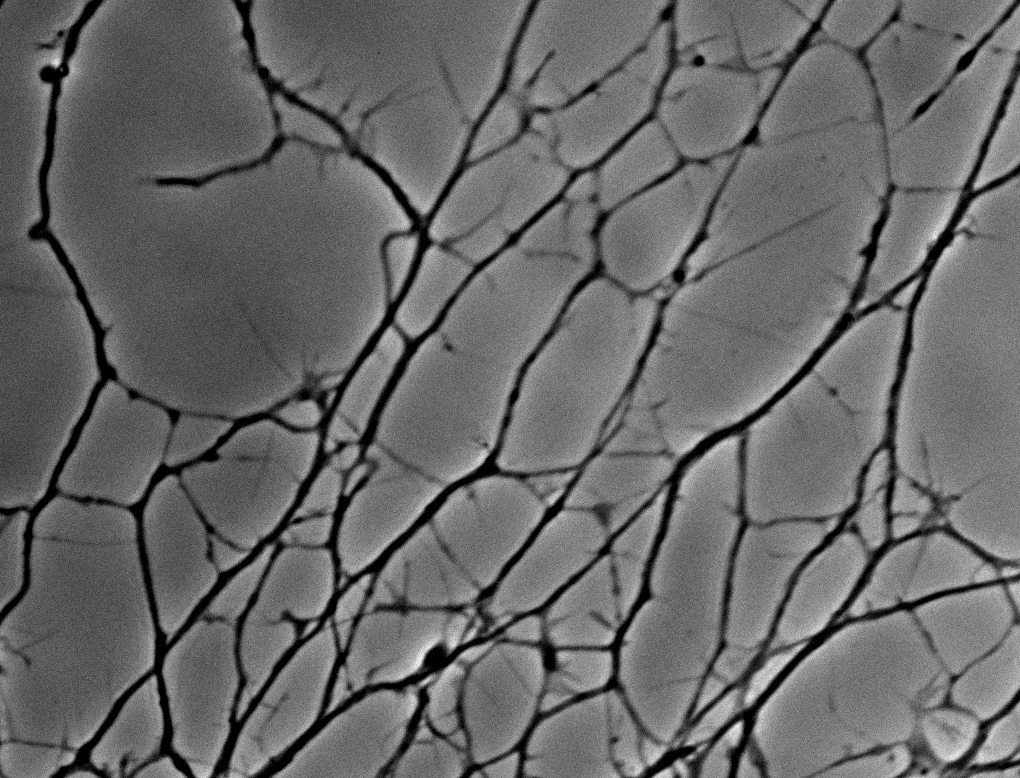

Supplement: Figure 6—source data 8. — The archive contains analyzed frames (TIFF format) and corresponding segmentation selection data (ImageJ-generated ZIP archives). Data can be displayed using ImageJ, please refer to Materials and methods. DOI: http://dx.doi.org/10.7554/eLife.19907.027 [file elife-19907-fig6-data8.zip › Figure_6_source_data_8/video15-60.tif]

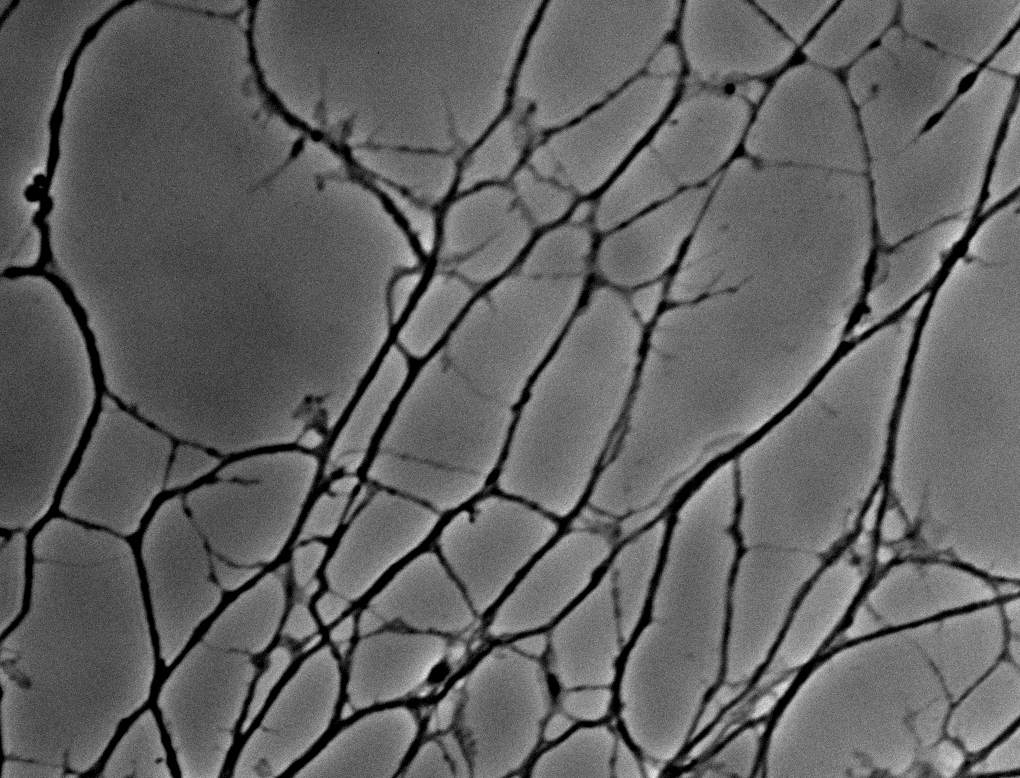

Supplement: Figure 6—source data 8. — The archive contains analyzed frames (TIFF format) and corresponding segmentation selection data (ImageJ-generated ZIP archives). Data can be displayed using ImageJ, please refer to Materials and methods. DOI: http://dx.doi.org/10.7554/eLife.19907.027 [file elife-19907-fig6-data8.zip › Figure_6_source_data_8/video15-70.tif]

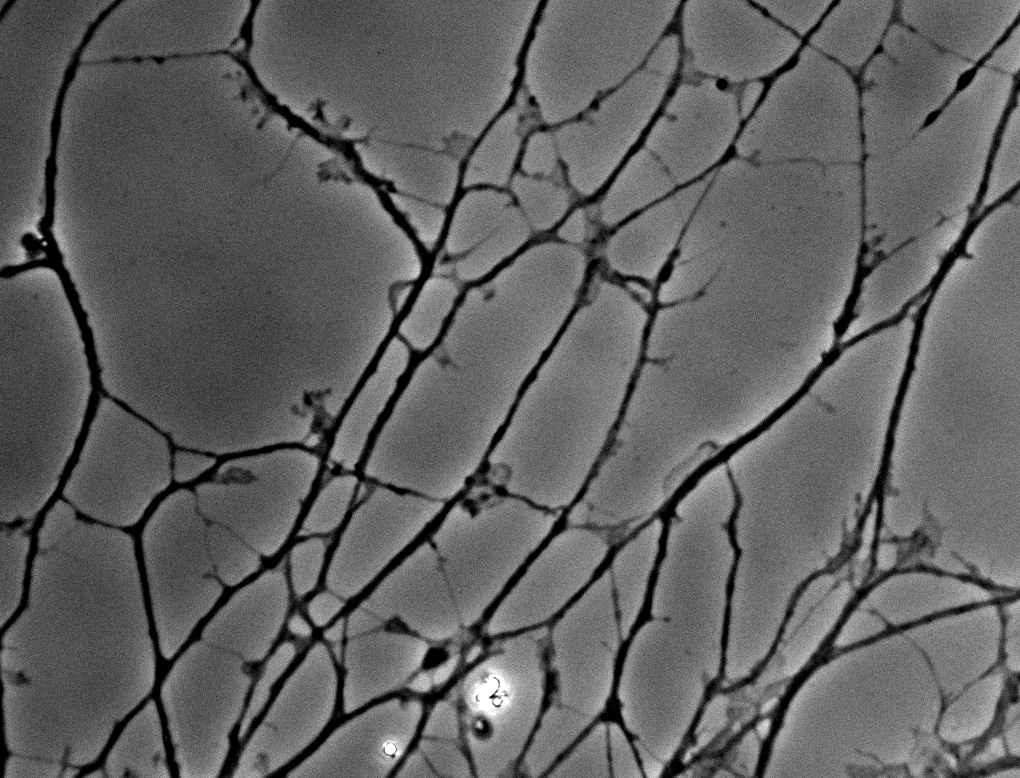

Supplement: Figure 6—source data 8. — The archive contains analyzed frames (TIFF format) and corresponding segmentation selection data (ImageJ-generated ZIP archives). Data can be displayed using ImageJ, please refer to Materials and methods. DOI: http://dx.doi.org/10.7554/eLife.19907.027 [file elife-19907-fig6-data8.zip › Figure_6_source_data_8/video15-80.tif]

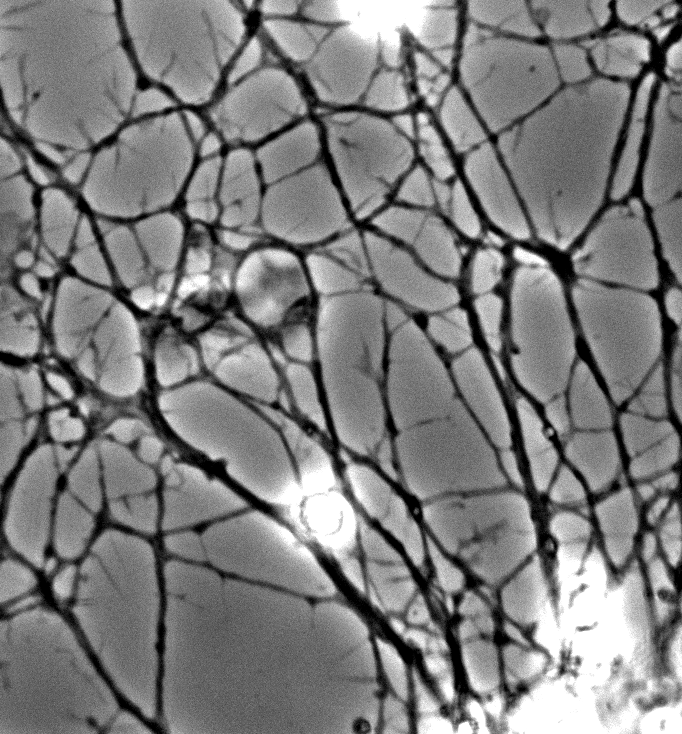

Supplement: Figure 6—source data 8. — The archive contains analyzed frames (TIFF format) and corresponding segmentation selection data (ImageJ-generated ZIP archives). Data can be displayed using ImageJ, please refer to Materials and methods. DOI: http://dx.doi.org/10.7554/eLife.19907.027 [file elife-19907-fig6-data8.zip › Figure_6_source_data_8/video16-1.tif]

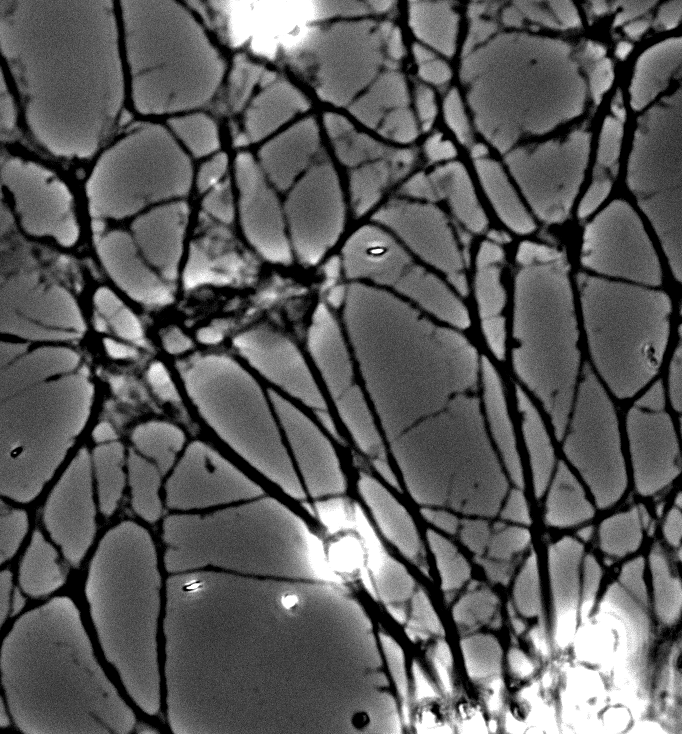

Supplement: Figure 6—source data 8. — The archive contains analyzed frames (TIFF format) and corresponding segmentation selection data (ImageJ-generated ZIP archives). Data can be displayed using ImageJ, please refer to Materials and methods. DOI: http://dx.doi.org/10.7554/eLife.19907.027 [file elife-19907-fig6-data8.zip › Figure_6_source_data_8/video16-100.tif]

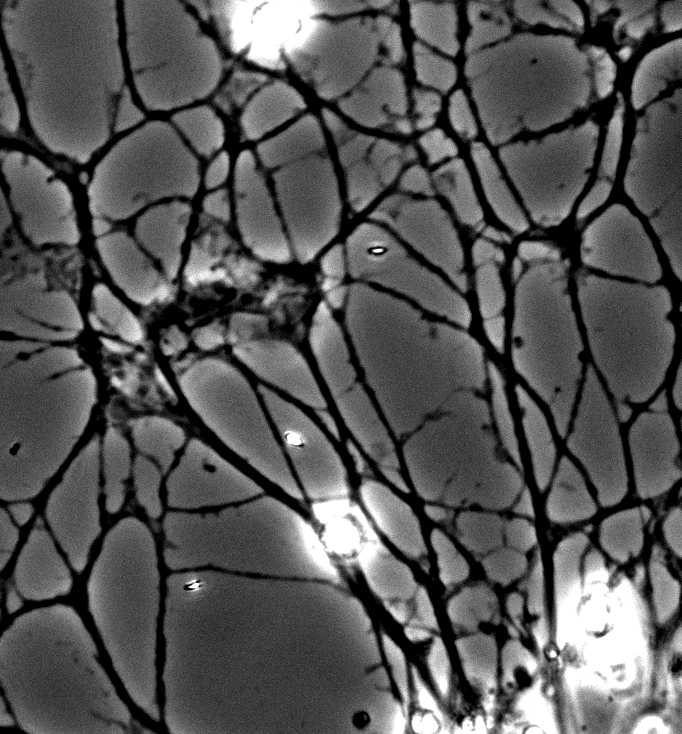

Supplement: Figure 6—source data 8. — The archive contains analyzed frames (TIFF format) and corresponding segmentation selection data (ImageJ-generated ZIP archives). Data can be displayed using ImageJ, please refer to Materials and methods. DOI: http://dx.doi.org/10.7554/eLife.19907.027 [file elife-19907-fig6-data8.zip › Figure_6_source_data_8/video16-120.tif]

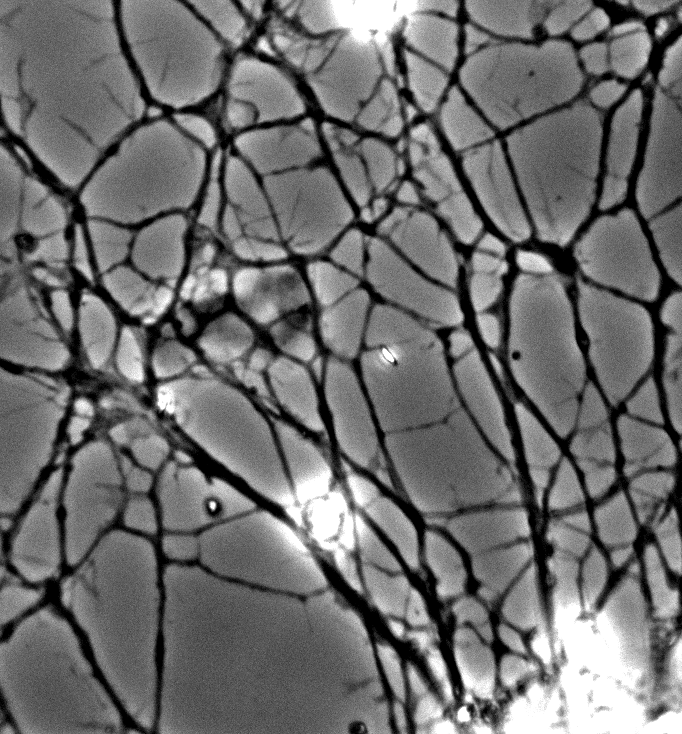

Supplement: Figure 6—source data 8. — The archive contains analyzed frames (TIFF format) and corresponding segmentation selection data (ImageJ-generated ZIP archives). Data can be displayed using ImageJ, please refer to Materials and methods. DOI: http://dx.doi.org/10.7554/eLife.19907.027 [file elife-19907-fig6-data8.zip › Figure_6_source_data_8/video16-30.tif]

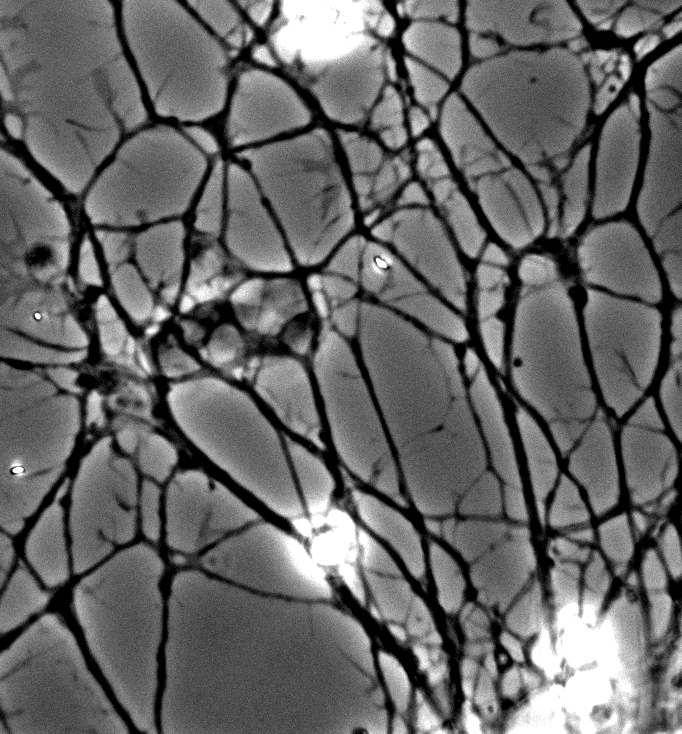

Supplement: Figure 6—source data 8. — The archive contains analyzed frames (TIFF format) and corresponding segmentation selection data (ImageJ-generated ZIP archives). Data can be displayed using ImageJ, please refer to Materials and methods. DOI: http://dx.doi.org/10.7554/eLife.19907.027 [file elife-19907-fig6-data8.zip › Figure_6_source_data_8/video16-50.tif]

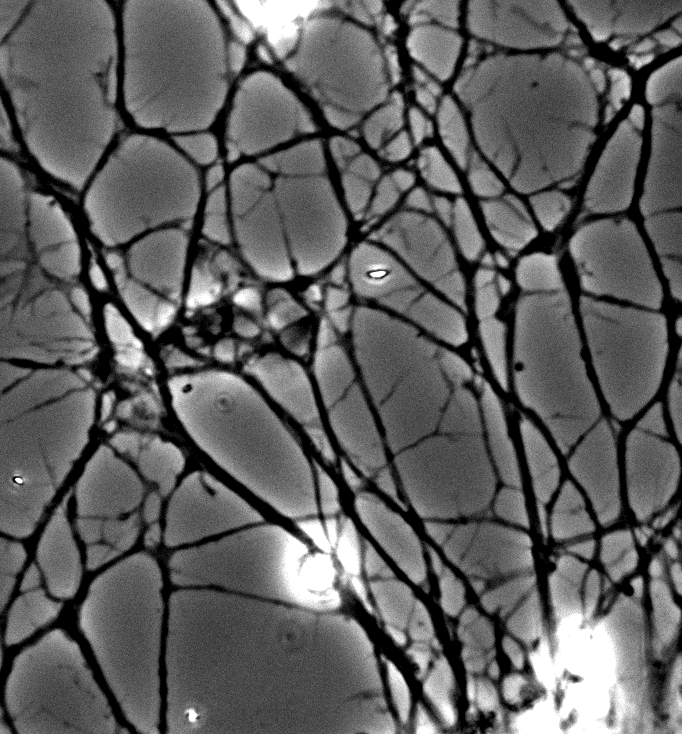

Supplement: Figure 6—source data 8. — The archive contains analyzed frames (TIFF format) and corresponding segmentation selection data (ImageJ-generated ZIP archives). Data can be displayed using ImageJ, please refer to Materials and methods. DOI: http://dx.doi.org/10.7554/eLife.19907.027 [file elife-19907-fig6-data8.zip › Figure_6_source_data_8/video16-66.tif]

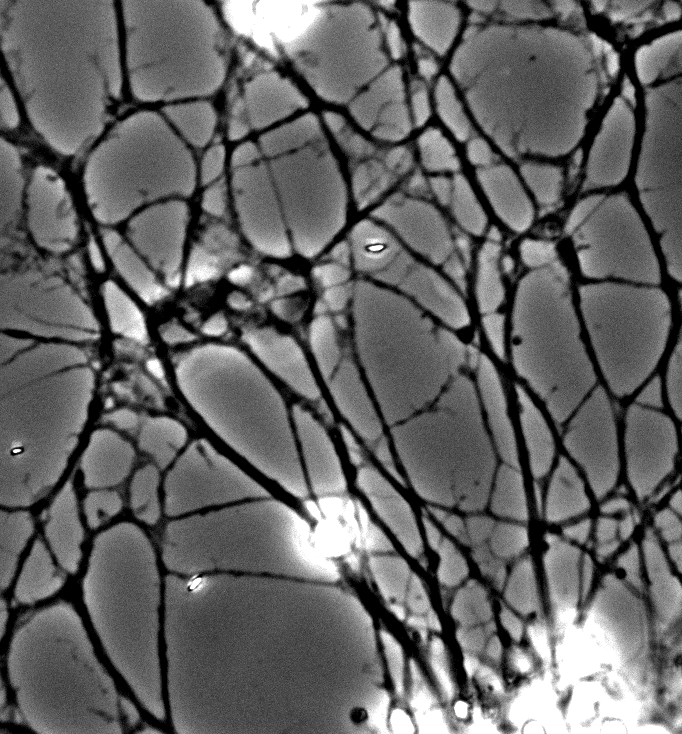

Supplement: Figure 6—source data 8. — The archive contains analyzed frames (TIFF format) and corresponding segmentation selection data (ImageJ-generated ZIP archives). Data can be displayed using ImageJ, please refer to Materials and methods. DOI: http://dx.doi.org/10.7554/eLife.19907.027 [file elife-19907-fig6-data8.zip › Figure_6_source_data_8/video16-75.tif]

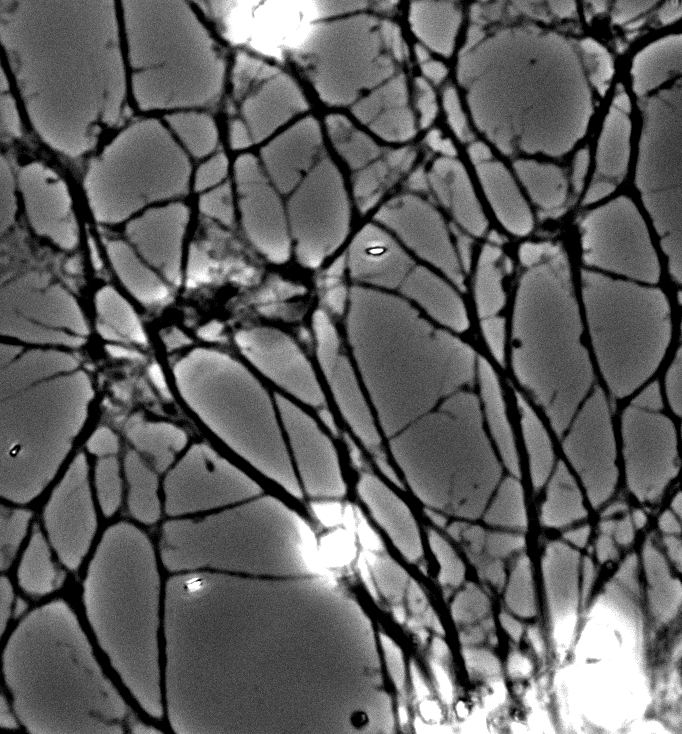

Supplement: Figure 6—source data 8. — The archive contains analyzed frames (TIFF format) and corresponding segmentation selection data (ImageJ-generated ZIP archives). Data can be displayed using ImageJ, please refer to Materials and methods. DOI: http://dx.doi.org/10.7554/eLife.19907.027 [file elife-19907-fig6-data8.zip › Figure_6_source_data_8/video16-85.tif]

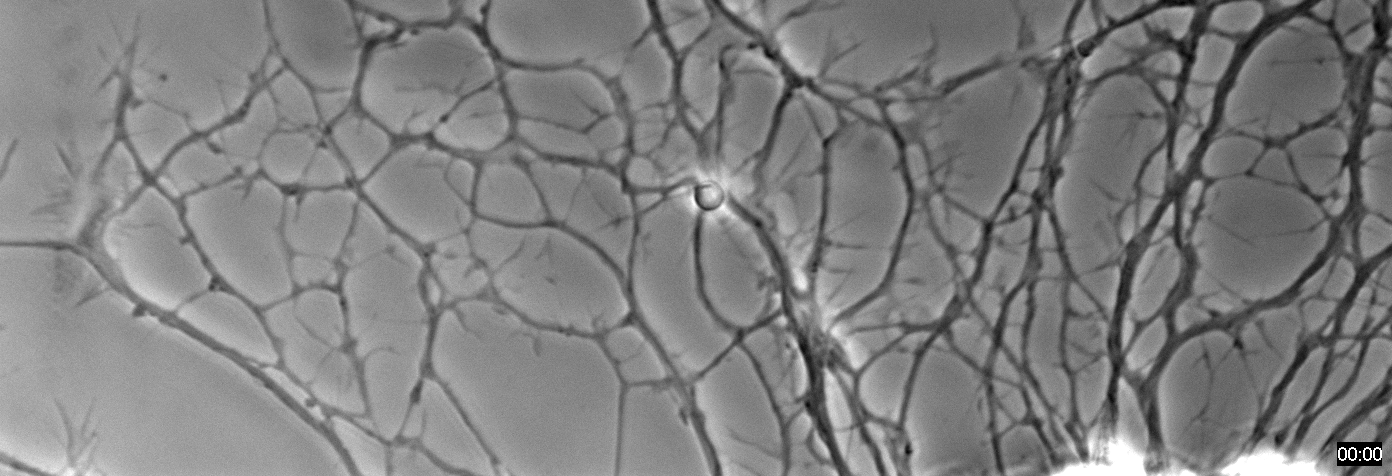

Supplement: Figure 6—source data 8. — The archive contains analyzed frames (TIFF format) and corresponding segmentation selection data (ImageJ-generated ZIP archives). Data can be displayed using ImageJ, please refer to Materials and methods. DOI: http://dx.doi.org/10.7554/eLife.19907.027 [file elife-19907-fig6-data8.zip › Figure_6_source_data_8/video17-1.tif]

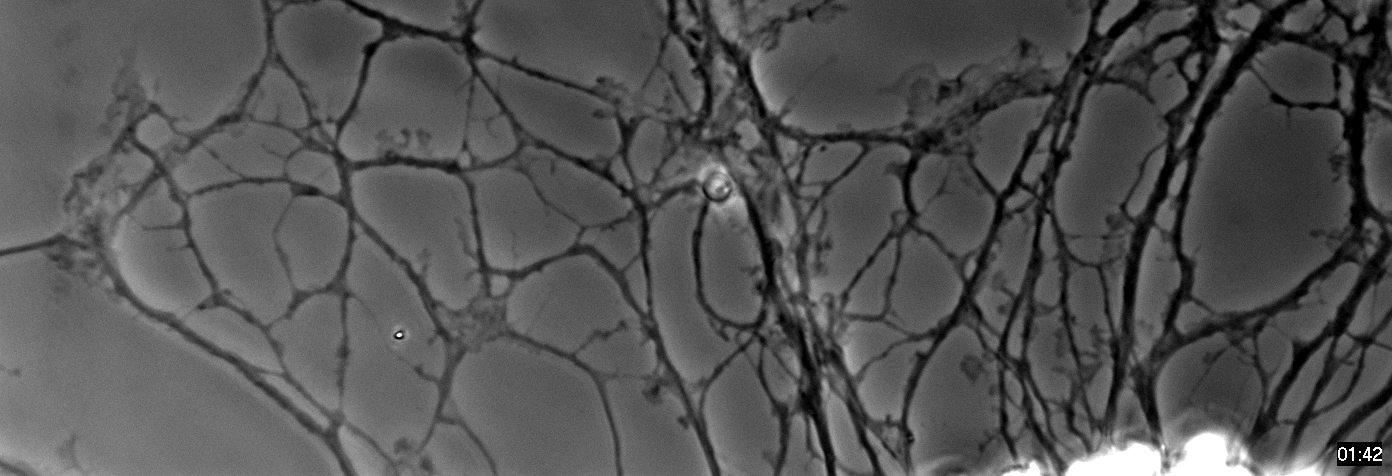

Supplement: Figure 6—source data 8. — The archive contains analyzed frames (TIFF format) and corresponding segmentation selection data (ImageJ-generated ZIP archives). Data can be displayed using ImageJ, please refer to Materials and methods. DOI: http://dx.doi.org/10.7554/eLife.19907.027 [file elife-19907-fig6-data8.zip › Figure_6_source_data_8/video17-100.tif]

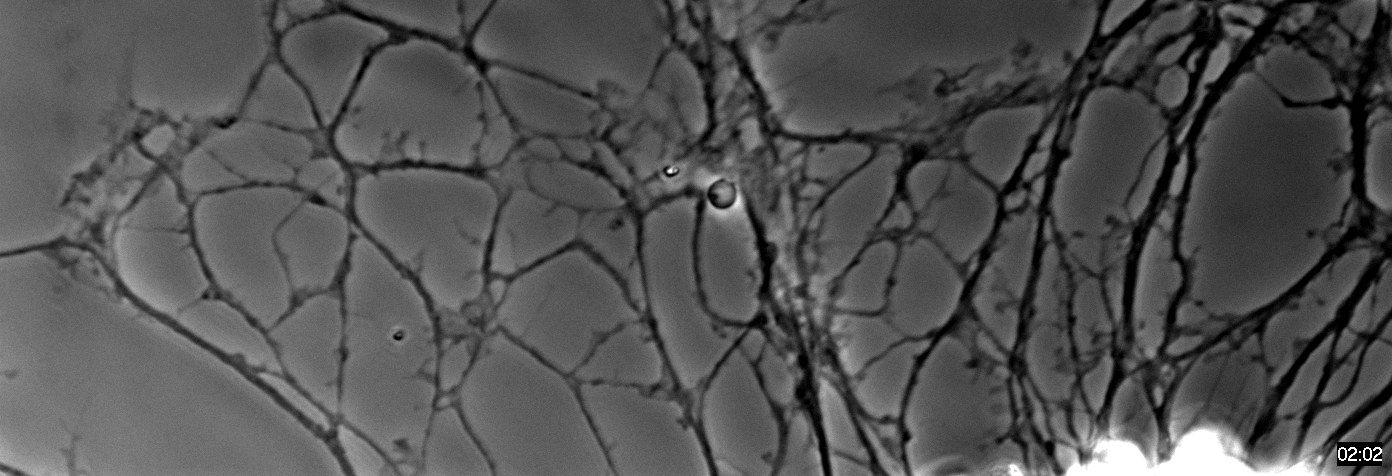

Supplement: Figure 6—source data 8. — The archive contains analyzed frames (TIFF format) and corresponding segmentation selection data (ImageJ-generated ZIP archives). Data can be displayed using ImageJ, please refer to Materials and methods. DOI: http://dx.doi.org/10.7554/eLife.19907.027 [file elife-19907-fig6-data8.zip › Figure_6_source_data_8/video17-120.tif]

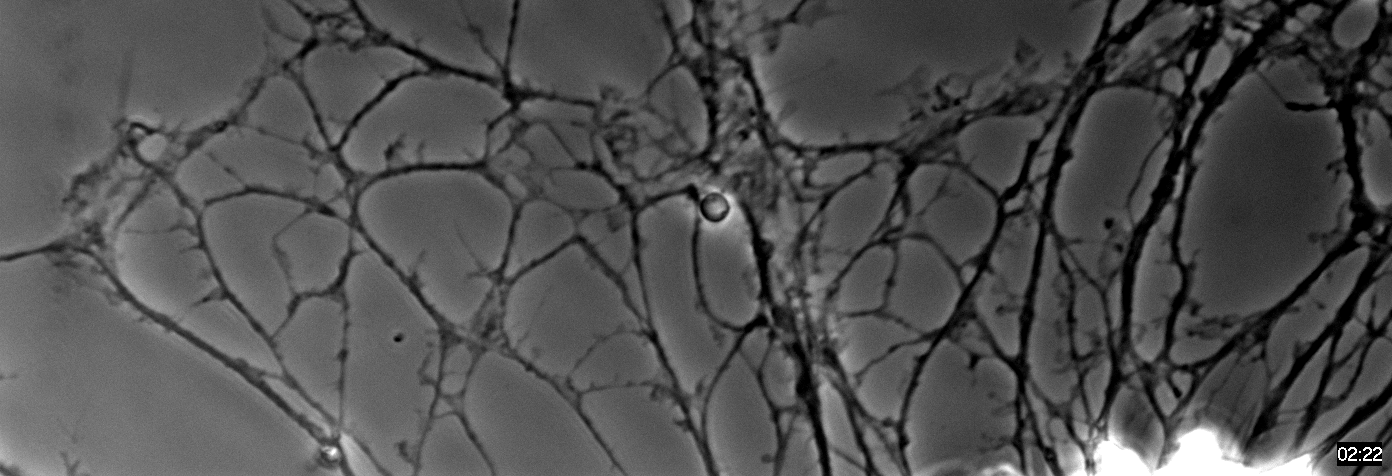

Supplement: Figure 6—source data 8. — The archive contains analyzed frames (TIFF format) and corresponding segmentation selection data (ImageJ-generated ZIP archives). Data can be displayed using ImageJ, please refer to Materials and methods. DOI: http://dx.doi.org/10.7554/eLife.19907.027 [file elife-19907-fig6-data8.zip › Figure_6_source_data_8/video17-140.tif]

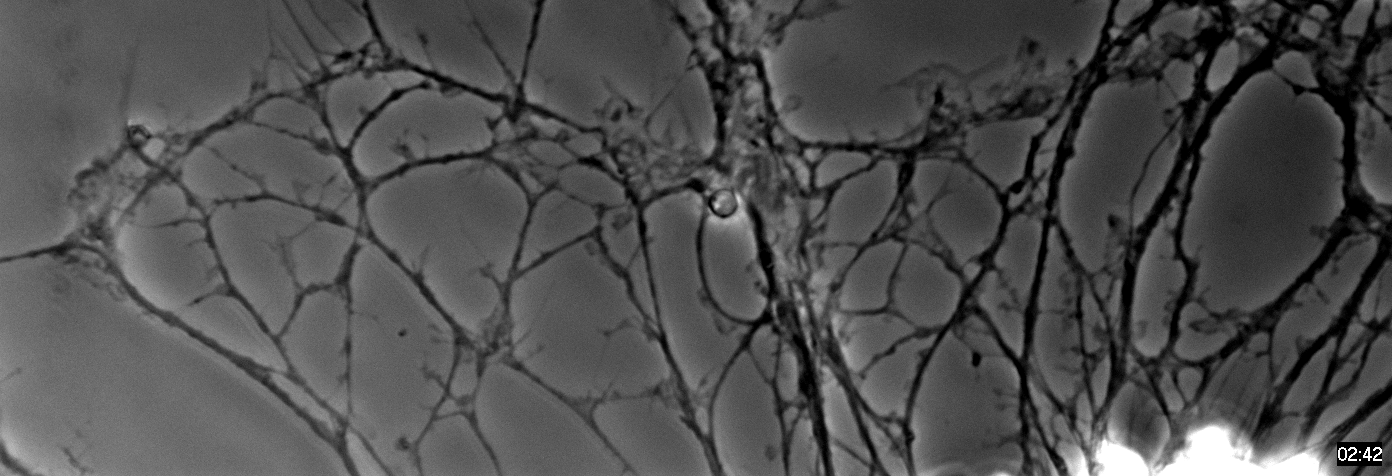

Supplement: Figure 6—source data 8. — The archive contains analyzed frames (TIFF format) and corresponding segmentation selection data (ImageJ-generated ZIP archives). Data can be displayed using ImageJ, please refer to Materials and methods. DOI: http://dx.doi.org/10.7554/eLife.19907.027 [file elife-19907-fig6-data8.zip › Figure_6_source_data_8/video17-160.tif]

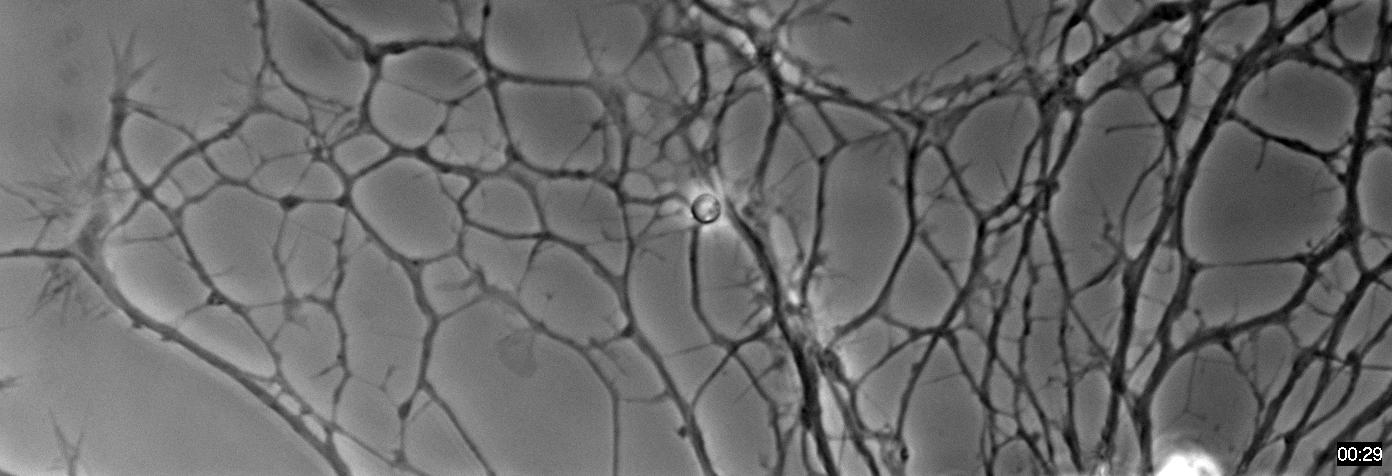

Supplement: Figure 6—source data 8. — The archive contains analyzed frames (TIFF format) and corresponding segmentation selection data (ImageJ-generated ZIP archives). Data can be displayed using ImageJ, please refer to Materials and methods. DOI: http://dx.doi.org/10.7554/eLife.19907.027 [file elife-19907-fig6-data8.zip › Figure_6_source_data_8/video17-30.tif]

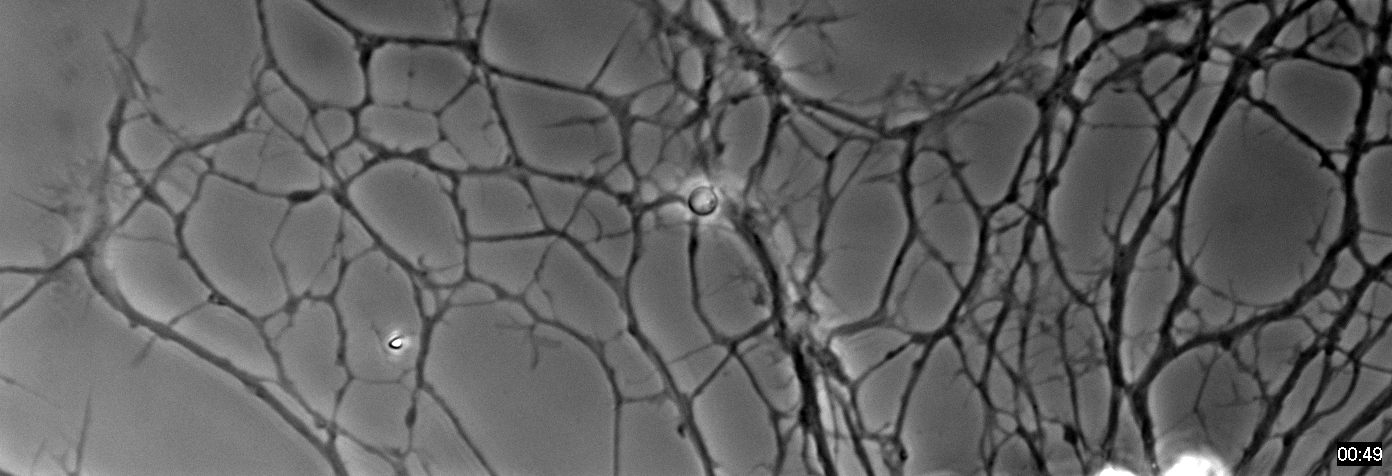

Supplement: Figure 6—source data 8. — The archive contains analyzed frames (TIFF format) and corresponding segmentation selection data (ImageJ-generated ZIP archives). Data can be displayed using ImageJ, please refer to Materials and methods. DOI: http://dx.doi.org/10.7554/eLife.19907.027 [file elife-19907-fig6-data8.zip › Figure_6_source_data_8/video17-50.tif]

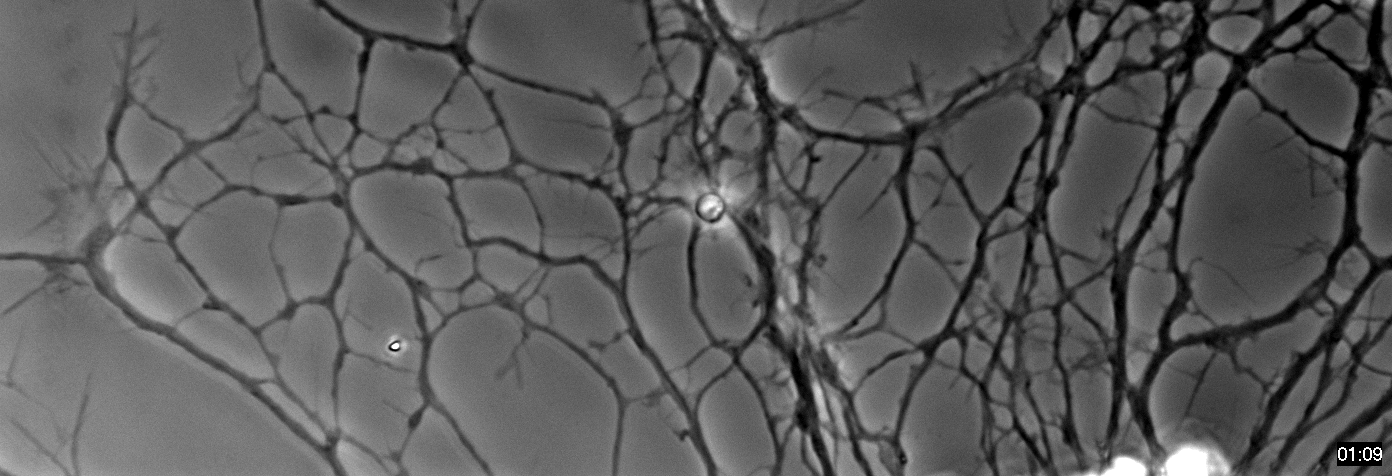

Supplement: Figure 6—source data 8. — The archive contains analyzed frames (TIFF format) and corresponding segmentation selection data (ImageJ-generated ZIP archives). Data can be displayed using ImageJ, please refer to Materials and methods. DOI: http://dx.doi.org/10.7554/eLife.19907.027 [file elife-19907-fig6-data8.zip › Figure_6_source_data_8/video17-70.tif]

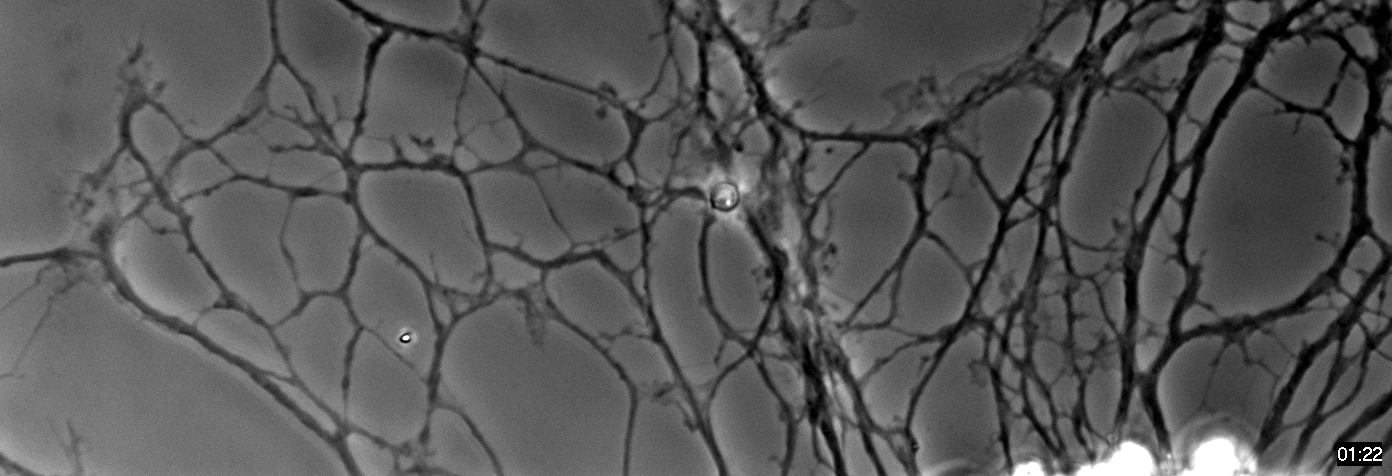

Supplement: Figure 6—source data 8. — The archive contains analyzed frames (TIFF format) and corresponding segmentation selection data (ImageJ-generated ZIP archives). Data can be displayed using ImageJ, please refer to Materials and methods. DOI: http://dx.doi.org/10.7554/eLife.19907.027 [file elife-19907-fig6-data8.zip › Figure_6_source_data_8/video17-80.tif]

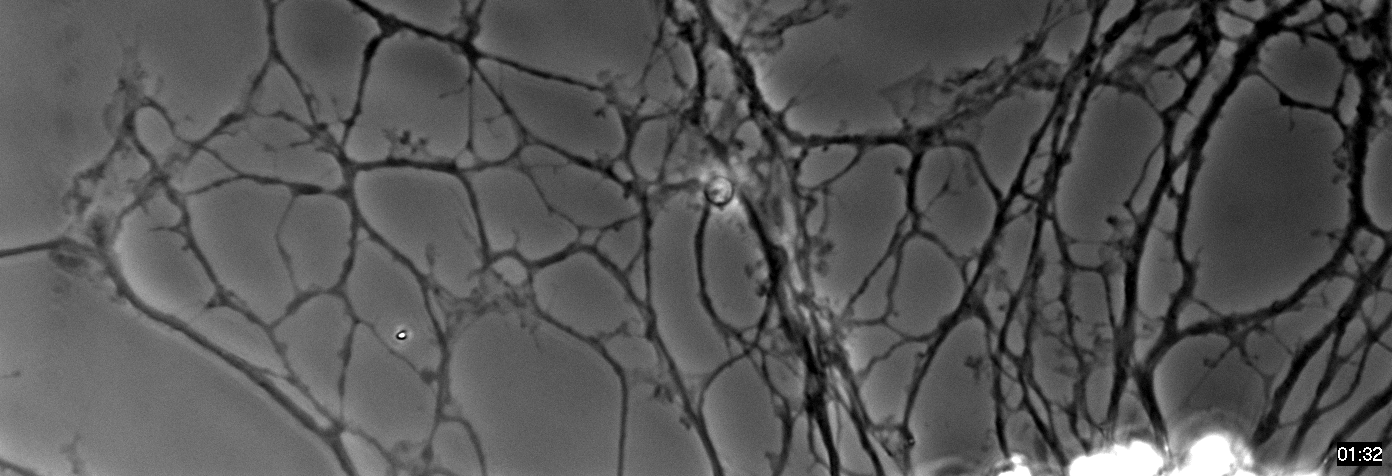

Supplement: Figure 6—source data 8. — The archive contains analyzed frames (TIFF format) and corresponding segmentation selection data (ImageJ-generated ZIP archives). Data can be displayed using ImageJ, please refer to Materials and methods. DOI: http://dx.doi.org/10.7554/eLife.19907.027 [file elife-19907-fig6-data8.zip › Figure_6_source_data_8/video17-90.tif]
